# Identification of Pathogenic Pathways for Recurrence of Focal Segmental Glomerulosclerosis after Kidney Transplantation

**DOI:** 10.3390/diagnostics14151591

**Published:** 2024-07-24

**Authors:** Sahra Pajenda, Daniela Gerges, Ludwig Wagner, David O’Connell, Monika Aiad, Richard Imre, Karl Mechtler, Alexander Zimprich, Alice Schmidt, Guerkan Sengoelge, Wolfgang Winnicki

**Affiliations:** 1 Department of Medicine III, Division of Nephrology and Dialysis, Medical University of Vienna, 1090 Vienna, Austria; sahra.pajenda@meduniwien.ac.at (S.P.); daniela.gerges@meduniwien.ac.at (D.G.); 1618704@students.meduniwien.ac.at (M.A.); alice.schmidt@meduniwien.ac.at (A.S.); guerkan.sengoelge@meduniwien.ac.at (G.S.); wolfgang.winnicki@meduniwien.ac.at (W.W.); 2 BiOrbic—Bioeconomy Research Centre, University College Dublin, Belfield, D04 V1W8 Dublin, Ireland; david.oconnell@ucd.ie; 3 School of Biomolecular and Biomedical Science, University College Dublin, Belfield, D04 V1W8 Dublin, Ireland; 4 ProtChem Facility, IMP-IMBA, Research Institute of Molecular Pathology—Institute of Molecular Biotechnology, 1030 Vienna, Austria; richard.imre@imp.ac.at (R.I.); karl.mechtler@imp.ac.at (K.M.); 5 Department of Neurology, Medical University of Vienna, 1090 Vienna, Austria; alexander.zimprich@meduniwien.ac.at

**Keywords:** focal segmental glomerulosclerosis (FSGS), kidney transplantation, recurrent FSGS, exome sequencing, expression analysis, protein array screening, pathway and gene enrichment analysis, alpha-2 macroglobulin (A2M), IL-17, CCL2/MCP-1

## Abstract

Primary focal segmental glomerulosclerosis (FSGS) is a disease of the podocytes and glomerulus, leading to nephrotic syndrome and progressive loss of renal function. One of the most serious aspects is its recurrence of disease in over 30% of patients following allogeneic kidney transplantation, leading to early graft loss. This research investigates the individual genetic predispositions and differences in the immune responses leading to recurrence of FSGS after transplantation. We performed exome sequencing on six patients with recurrent FSGS to identify variants in fifty-one genes and found significant variations in the alpha-2-macroglobulin (A2M). Immunoblotting was used to investigate effects of specific gene variants at the protein level. Further expression analysis identified A2M, exophilin 5 (EXPH5) and plectin (PLEC) as specific proteins linked to podocytes, endothelial cells, and the glomerulus. Subsequent protein array screening revealed the presence of non-HLA-specific antibodies, including TRIM21, after transplantation. Using Metascape for pathway and process enrichment analysis, we focused on the IL-17 signaling and chemotaxis pathways. ELISA measurements showed significantly elevated IL-17 levels in patients with recurrent FSGS (32.30 ± 9.12 pg/mL) compared to individuals with other glomerular diseases (23.16 ± 2.49 pg/mL; *p* < 0.01) and healthy subjects (22.28 ± 0.94 pg/mL; *p* < 0.01), with no significant difference in plasma CCL2/MCP-1 levels between groups. This study explores the molecular dynamics underlying recurrence of FSGS after transplantation, offering insights into potential biomarkers and therapeutic targets for the future development of individualized treatments for transplant patients.

## 1. Introduction

Kidney transplantation is the only effective therapy for end-stage renal diseases (ESRD) and leads to a significant improvement in morbidity and mortality. However, some glomerular diseases can recur in the transplanted organ, such as focal segmental glomerulosclerosis (FSGS). Therefore, a precise differentiation between primary and secondary FSGS is crucial, as the primary form in particular tends to recur in the kidney transplant in up to one third of recipients [1].

Understanding the underlying pathophysiologic mechanisms appears to be an important step to tackle this disease and its recurrence. The nephrin gene (NPHS1), which is specifically expressed in podocytes and localized in the slit diaphragm, has been shown to be associated with FSGS [2,3]. Zhuo et al. could delineate NPHS1 mutations in a significant subgroup of FSGS patients in China [4]. Initially unexpected, it was found that the recurrence of nephrotic syndrome after transplantation in patients with congenital nephrotic syndrome of Finnish-type was caused by the development of antibodies against the donor NPHS1 genotype [5]. Recent extensive work on the development of non-HLA antibodies after allogeneic kidney transplantation has made this observation more understandable [6]. In this regard, the intriguing observation was made that a kidney transplant that did not work in a recipient with primary FSGS immediately functioned after explantation and subsequent implantation in a second recipient with different underlying disease [7].

There has been an ongoing search for a disease-causing permeability factor [8] and potential candidates include cytokines as well as the soluble urokinase-type plasminogen activator receptor (suPAR) [9]. Although suPAR indicates an active disease process [10] and its cleaved product acts as chemotactic agent, a clear causal pathogenic association could not be attributed [11,12].

Glomerular endothelial cell dysfunction is considered to be the triggering factor leading to inadequate crosstalk with podocytes and progressive podocyte pathology [13]. This occurs as glomerular endothelial cells are the primary site of exposure to circulating pathogenic factors in the blood, which can alter the glycocalyx and affect stress signaling pathways within endothelial cells. In this context, the molecule alpha-2-macroglobulin (A2M) has been described via single-cell transcriptomics, which is expressed in glomerular endothelial cells and circulates in the blood [14]. A2M stands associated with steroid resistance in nephrotic patients and plays a central role in nephrotic syndrome [15] because its macromolecular size and structure prevents it from passing through the slit diaphragm [16], even if other macroproteins such as albumin are lost in the urine [17]. A2M’s upregulation and production in the liver in response to albumin loss indicates its importance in maintaining blood oncotic pressure.

In addition, mutations in several proteins of the cytoskeleton have been shown to be involved in the pathogenesis of FSGS. Such mutations disrupt the integrity of the podocyte cytoskeleton, resulting in damage to the podocytes, which are very sensitive to shear forces [18].

In this study, we present findings of patients with recurrence of FSGS after kidney transplantation with different response to established therapeutic measures:

First, we analyzed exome sequencing data to identify potentially harmful heterozygous genotypes. Subsequently, immunoblots were conducted to evaluate how these specific gene variants undergo protein-level modifications. Following expression analysis specific for kidney tissue, a protein array screening was performed to investigate the potential development of non-HLA-specific antibodies after kidney transplantation. To investigate whether downstream products of identified gene variants contributed to the development and recurrence of FSGS in the kidney transplant, a pathway and process enrichment analysis was conducted. Finally, pathway-specific end products were measured on the protein level using ELISA.

## 2. Materials and Methods

### 2.1. Study Design

The study, conducted in accordance with the Helsinki Declaration, received approval from the Ethics Committee of the Medical University of Vienna (EK 1465/2013). Written informed consent was obtained from the study participants, who were all over 18 years old. All procedures adhered to relevant guidelines and regulations. Funding for this research was provided by the Medical Scientific Fund of the Mayor of the City of Vienna, grant numbers 22099 and 22100.

The aim of this study was to identify possible causes for the recurrence of FSGS after kidney transplantation in patients with primary FSGS. Therefore, 6 patients with recurrence of FSGS in the kidney transplant, 45 patients with different glomerular diseases, and 31 healthy controls were included in the study. The primary samples were collected between June 2013 and April 2015. Patients with recurrence of FSGS are referred to as patients A, B, C, D, E, and F throughout the manuscript.

### 2.2. Data Collection and Exome Sequencing

EDTA blood samples collected from the six patients with recurrence of FSGS in the allograft were used for isolation of genomic DNA. The purified DNA was submitted for exome sequencing at the Technical University of Munich, Germany. Resulting data were integrated into the Helmholtz database comprising 19,900 individuals of various European origins and the data sets of the six study patients were compared by searching for missense mutations against this comprehensive reference dataset. Of particular interest were variants involving proteins expressed in human glomeruli. The glomerular expression pattern was verified using the Human Protein Atlas (https://www.proteinatlas.org/, accessed on 18 December 2023) and the Nephrocell database (http://nephrocell.miktmc.org/, accessed on 18 December 2023).

### 2.3. Expression Analysis

To identify the potential expression of mutated gene products in podocytes and endothelial cells, the Nephrocell database was searched in the subdomain “Adult normal kidney”. Here, the results are presented according to their distribution within 31 clusters, which represent specific gene expression patterns for individual cell types.

### 2.4. Immunoblotting

Five hundred nanolitres of serum were mixed with 1x sample buffer (BioRad 4x Laemmli sample buffer, Cat. #1610747). The samples were loaded onto a Mini Protean TGX gel and transferred onto nitrocellulose by semidry blotting. For kidney specific autoantibody immunoblotting renal protein extract was loaded onto a Mini Protean TGX gel (Cat. # 4561081) and correspondingly blotted. After blocking the filter with Pierce™ Protein-free T20 (TBS) blocking buffer, the membrane was incubated with monospecific rabbit anti A2M Ab (ab48555). After overnight incubation at 4 °C, the filter was incubated in HRP conjugated goat anti rabbit IgG-specific secondary antibody (Dako PO448; dil 1:10,000 in blocking buffer). For detecting human kidney specific autoantibodies, HRP conjugated rabbit anti human IgG-specific secondary antibody (Dako PO214; dil 1:10,000 in blocking buffer) was applied. Following 60 min of incubation at room temperature (RT), the antibody binding signal was developed using chemiluminescence reagent and recorded using the Fusion FX Vilber Lourmat (Vilber, Eberhardzell, Germany). Images were further processed using Photoshop version 6.0 (Adobe Inc., San Jose, CA, USA).

### 2.5. Protein Array Screening

Human protein microarrays (HuProtTM, Human Proteome Microarrays, Cambridge Protein Arrays) were tested at 4 °C, blocked for 60 min with 5% human serum albumin in Tris Buffered Saline and 0.1% Tween (TBST), and incubated for 1.5 h with 55-fold diluted human serum. After a 5 × 2-min TBST wash, an Alexa Fluor 642-labelled polyclonal goat anti-human specific Ab (A21445, Invitrogen, Waltham, MA, USA) was incubated at a concentration of 1 µg/mL for 60 min, followed by a further 5 × 2-min TBST wash. The microarrays were then rinsed, dried, and imaged on a Genepix 4000B scanner (Axon Instruments, San Jose, CA, USA) with PMT gain settings of 450 for the 635 nm laser. A batch-specific gal file was used to generate .gpr result files from the scans and these files were analyzed using a custom software script.

### 2.6. Pathway and Gene Enrichment Analysis

For pathway and enrichment process analysis, Metascape was used [19]. All 25,000 genes were used as the enrichment background. The enrichment factor was calculated, which represents the ratio between the observed counts and the randomly expected counts. Enrichment-terms with a *p*-value < 0.01, a minimum count of 3, and an enrichment factor > 1.5 were collected and grouped into clusters based on their similarities. More specifically, *p*-values were calculated based on the cumulative hypergeometric distribution, and q-values were calculated using the Benjamini–Hochberg procedure to account for multiple testing [20]. Kappa scores [21] were used as the similarity metric when performing hierarchical clustering on the enriched terms, and sub-trees with a similarity of >0.3 were considered a cluster. The most statistically significant term within a cluster was chosen to represent the cluster.

### 2.7. IL-17 Analysis by ELISA

The IL-17 ELISA was purchased from RD Quantikine RD/QK317 and the procedure given in the test manual was followed. Briefly, 50 µL of sample or standard was added to each well. After preparation of the antibody cocktail supplied with the test kit, 50 µL was applied to each well. The test was incubated in the foil-covered plate on a rotating platform for 60 min at RT. The assay was then washed with the supplied wash buffer using an ELISA plate washer and the chromogenic substrate was added to each well and incubated for 20 min at RT under light protection. Finally, 50 µL of stop solution was applied and the ELISA test was read at 450 nm using an ELISA plate reader. The concentrations were calculated using the standards included on the ELISA plate.

### 2.8. CCL2/MCP-1 Analysis by ELISA

The chemokine ligand 2 (CCL2)/monocyte chemoattractant protein-1 (MCP-1) ELISA was performed as indicated in the test manual. After adding 50 µL of the RD1-83 test diluent to each well, the pre-diluted plasma (1:2) was applied together with the standard series and incubated for 2 h at RT. The washing procedure was performed with an automatic ELISA washing machine using the washing buffer provided in the test kit. The anti-human MCP-1 conjugate was then added (200 µL) and incubated again for 2 h at RT on a shaking platform. After the second wash, the substrate/chromogen mixture was applied and incubated under light protection for 30 min at RT. The ELISA was then read at 450 nm using the ELISA reader after addition of the stop solution and sample concentrations were calculated using the standard curve included on each plate.

### 2.9. Routine Laboratory Parameters and Statistical Analysis

All laboratory parameters including renal function values were determined at the Department of Laboratory Medicine of the Medical University of Vienna with certified (ISO 9001:2008) [22] and accredited (ISO 15189:2008) [23] quality management system. Routine data and treatment measures were extracted from the clinical data base, and data management, analysis and processing were performed using Microsoft Excel for Windows (Microsoft, Redmond, WA, USA), Photoshop version 6.0 (Adobe Inc.; San Jose, CA, USA), and GraphPad Prism 8 (Dotmatics, Boston, MA, USA). Categorized data are shown as absolute number and relative frequency. Continuous data are presented as mean ± standard deviation. For comparison of non-normally distributed variables, Mann–Whitney U test was utilized. A *p*-value less than 0.05 was considered statistically significant.

## 3. Results

### 3.1. Patient Demographics and Clinical Course of Study Patients

The study aimed to investigate and identify potential causative factors driving FSGS and its recurrence in the transplanted kidney. At the time of the study, patients with recurrence of FSGS were 28–57 years old and had a biopsy-proven recurrence of disease after transplantation. Their immunosuppressive treatment regimens included prednisolone, mycophenolate mofetil (MMF), tacrolimus (Tac), cyclosporine A (CyA), rituximab (RTX), plasma exchange (PLEX), immunoapheresis, and abatacept. The individual clinical course and treatment interventions for all patients with recurrence of FSGS after kidney transplantation are shown in File S1.

### 3.2. Exome Sequencing

Exome sequencing results of patients with recurrence of FSGS in the transplant were entered into the Helmholtz database and the datasets were compared by searching for missense mutations in any gene occurring less than 15 times among 19,900 donor exomes. By setting this algorithm, 51 genes with missense mutations were found with non-synonymous single nucleotide polymorphisms (nsSNPs), causing an amino acid change and thereby predicting one of the following outcomes: benign, possibly dangerous, or damaging for the function of the protein (Table 1).

Among the rare denoted gene mutations listed in Table 1, three out of six patients with recurrence of FSGS in the allograft (patients A, B, and C) were found to have a missense mutation in the A2M gene (Table 2). The mutation in patient B was rated as “probably damaging” by the Polymorphism Phenotyping v2 prediction (PolyPhen-2, pph2) of functional effects of human non-synonymous single nucleotide polymorphisms (nsSNPs). The potential structure-altering property results from its location about 38 Å away from the cysteine of the thiol-ester domain 1 CGEQ 972-975, the functional unit of this protein, according to the protein data bank (PDB) structure of the native A2M monomer (7VON) (Figure S1).

In contrast, the mutations in patients A and C were classified as benign by pph2 (Table 2). Given the high variation of the A2M protein in our collective, we performed an in-depth study of the protein behavior in serum biology. This was of particular interest as the A2M protein plays a central role in nephrotic patients with hypoalbuminemia by preserving intravascular oncotic pressure as it does not pass through the damaged slit diaphragm due to its high molecular weight [17].

The “probably damaging” missense mutation of Glu1165Gly in patient B located near the end of the TED represents the functional site of the protease inhibitor [16]. To determine whether this could lead to changes in A2M aggregate formation and potential protease binding, immunoblots were performed under non-reducing conditions from serum of patient B and compared to serum from a verified normal A2M donor (Figure S2) at two different time points and disease status. The largest difference was observed in the high molecular weight aggregate bands. While the migration of the primary A2M band remained largely unchanged, there was a remarkable difference of the migratory pattern of the larger aggregates, highlighted by arrows in Figure S2 (B_1_, B_2_) in patient B with the mutation close to the thioester domain. These aggregates disappeared when EDTA was added to the sample buffer and the sample was heated to 95 °C before loading; furthermore, such high molecular weight bands were not observed when A2M was assayed in plasma by immunoblotting.

**Figure 1:**
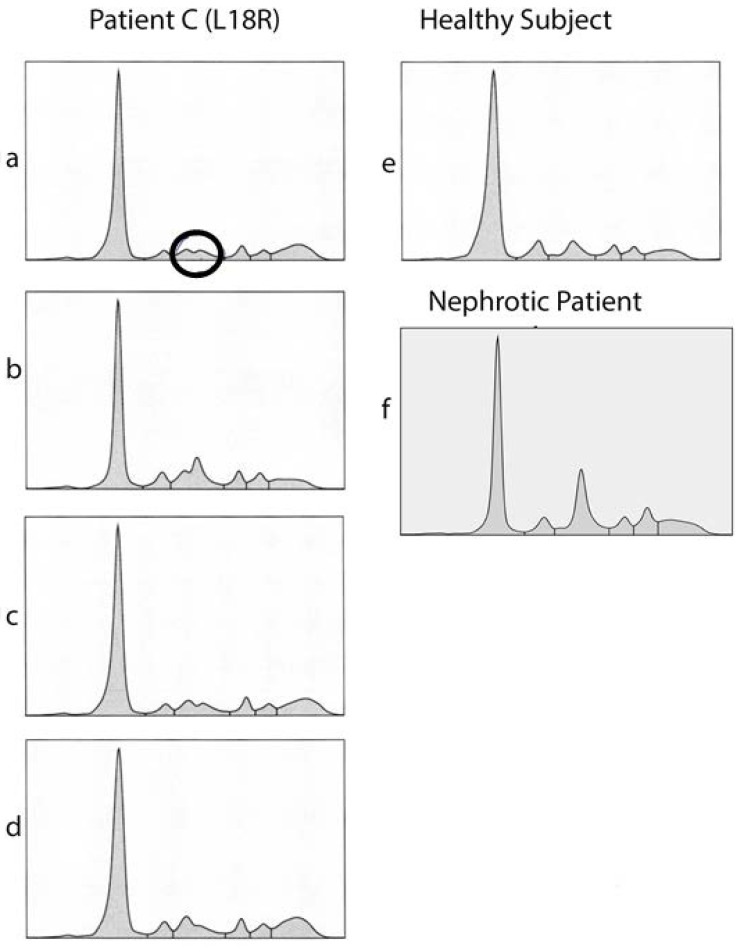
Serum Electrophoresis of Patient with Heterozygous Leu18Arg Missense Mutation in A2M. The morphology of the double peak formation in the alpha2 fraction (marked as circle in **a**) changed over time and course of disease (**a**–**d**). For comparison, the serum electrophoresis of a healthy subject (**e**) and of a patient with nephrotic syndrome (**f**). Data were recorded by capillary electrophoresis; the *x*-axis represents capillary mobility and the *y*-axis abundance of protein.

Given the second peak in the serum electrophoresis is mainly composed of the A2M protein, our focus turned to studying the A2M protein in patient C, who had a Leu18Arg missense mutation within the signal peptide of the protein (Figure 1). Of interest, we noted a patient-specific, unusual bipartite alpha-2 fraction in the serum electrophoresis (marked as circle in Figure 1a), which changed its distribution over the course of the disease (Figure 1a–d). In this context, the second peak was initially larger, but later leveled off as proteinuria decreased. For comparison, serum electrophoresis of a healthy donor and of a nephrotic patient are depicted in Figure 1e,f, respectively.

### 3.3. Expression Analysis

To characterize the expression pattern of the identified altered gene products, all 51 genes were entered into the Nephrocell database server in the subdomain “adult normal kidney” to allow differentiation into 31 clusters identified within the nephron (http://nephrocell.miktmc.org/, accessed on 18 December 2023).

Among the 51 genes, 3 exhibited remarkable specificity, being prominently expressed either in podocytes or closely associated endothelial cells and the glomerulus, namely A2M, exophilin 5 (EXPH5), and plectin (PLEC) (Figure S3).

The A2M gene product was found in several clusters: arteriolar endothelial cells, peritubular endothelial cells, and glomerular capillary endothelial cells as well as vascular smooth muscle and mesangial cells and monocytes (Figure S3a).

The EXPH5 gene expression was found exclusively in podocytes (Figure S3b). Here, patient D had a Gly1887Glu mutation graded “probably dangerous”, while patient E carried a Ser1169Pro variant classified as “benign”.

PLEC was found in arteriolar endothelial cells (Figure S3c). Here, patient D carried an Ala3337Val variant classified as “benign”, whereas patient F had a Glu1646Lys mutation graded as “probably damaging”.

### 3.4. Protein Array Screening

To investigate the potential development of non-HLA-specific antibodies after allograft transplantation, the sera of all six patients with recurrence of disease following kidney transplantation were examined using a multilane immunoblot on which renal proteins were immobilized. Among these six patients, patient E displayed a distinctive protein band detection pattern. Additionally, patient E developed rapid recurrence of FSGS after transplantation, leading to allograft loss despite intensified immunosuppressive treatments (File S1). Subsequently, patient E underwent screening for serum antibodies against 16,000 individual human proteins using established microarray technology (HuProtTM v4.0) [24]. This screening revealed a serum IgG antibody binding strongly to the tripartite motif containing 21 (TRIM21) protein, identified as the second strongest signal among all others (Table 3). Notably, patient E carried a heterozygous germline mutation (Phe446Ile) in TRIM21 classified as “potentially dangerous”. This observation is particularly noteworthy given the involvement of TRIM21 in immune regulation and response to viral invasions.

### 3.5. Pathway and Gene Enrichment Analysis

**Figure 2:**
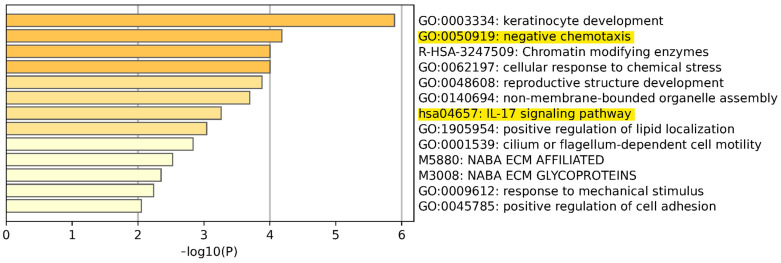
Metascape enrichment analysis of 51 altered genes linked to recurrence of FSGS in kidney allografts. The analysis identified 13 clusters, each represented by significant enrichment terms.

To determine whether downstream products of the 51 altered genes identified through exosome sequencing could contribute to the development and recurrence of FSGS in the kidney allograft, a pathway and process enrichment analysis was performed using the Molecular Signature Database Metascape. This analysis utilized ontology sources such as KEGG Pathway, GO Biological Processes, Reactome Gene Sets, Canonical Pathways, CORUM, WikiPathways, and PANTHER Pathway. As a result, 13 clusters were identified, each with representative enrichment terms (Figure 2). The comprehensive search results are provided in Table S1, which includes the GEO terms of all the listed genes. Employing this search strategy, the most promising gene sets revealed enrichment in the IL-17 signaling and chemotaxis pathways.

### 3.6. IL-17 Signaling Pathway

IL-17 is a cytokine involved in the regulation of immune responses, particularly in inflammation. It is mainly produced by a subset of T cells and plays a central role in the pathogenesis of various autoimmune diseases by promoting inflammation and tissue damage [25]. These include psoriasis [26], rheumatoid arthritis [27], multiple sclerosis [28], inflammatory bowel disease [29], systemic lupus erythematosus [30,31], graft versus host disease in bone marrow transplant recipients [32], as well as glomerular diseases [33,34]. In FSGS, IL-17 may contribute to pathogenesis by promoting inflammation and immune-mediated injury within the glomeruli, leading to podocyte apoptosis and injury [35].

Several gene mutations involved in the IL-17 signaling pathway were identified in our patients, with a number of them considered probably or possibly dangerous, having an impact on the biological function of the gene product (Table S2).

**Figure 3:**
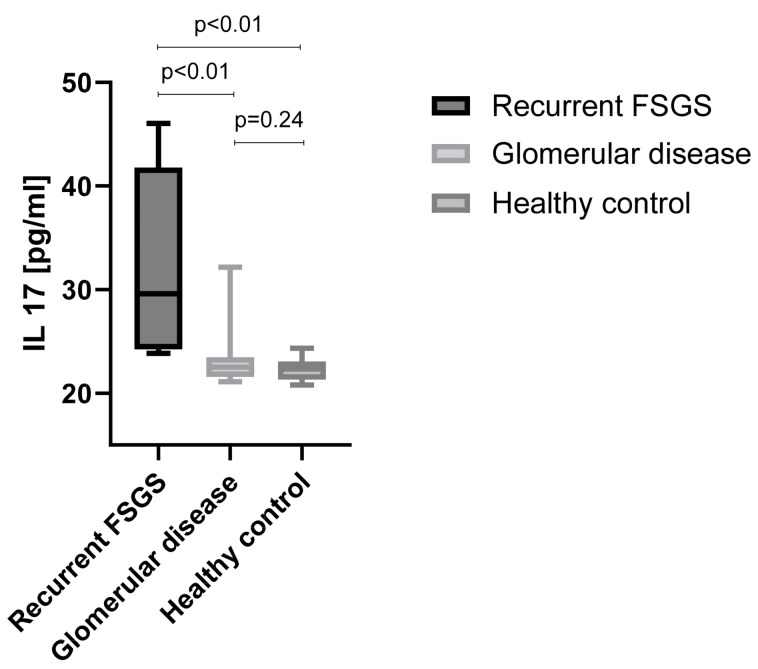
Plasma concentration of IL-17 in patients with FSGS recurrence after kidney transplantation compared to patients with other glomerular disease and healthy individuals.

Therefore, to test for differences at the protein level, IL-17 levels were measured in kidney transplant recipients with recurrence of FSGS, in patients with other glomerular diseases, and in healthy individuals by commercially available ELISA. This revealed increased plasma IL-17 levels in patients with recurrence of FSGS (32.30 ± 9.12 pg/mL; *n* = 6) versus glomerular disease (23.16 ± 2.49 pg/mL; *p* < 0.01; *n* = 45) and healthy subjects (22.28 ± 0.94 pg/mL; *p* < 0.01; n = 26) (Figure 3).

### 3.7. Chemotaxis Pathway

Several inflammatory cytokines are involved in the state of microinflammation observed in various renal diseases. Both immune cells and intrinsic renal cells, including podocytes, secrete proinflammatory cytokines such as IL-1, IL-6, TNF-alpha, and MCP-1. These cytokines take a central role in maintaining the inflammatory cascade and can contribute to the progression of the disease. In diseases such as diabetic nephropathy, the induction of MCP-1 and keratinocyte chemoattractant [36] has been associated with increased podocyte death, highlighting the importance of these inflammatory mediators in renal pathology [37].

In our patient cohort, we could identify several gene variants involved in the chemotaxis pathway, with many mutations designated as probably or possibly damaging (Table S3).

**Figure 4:**
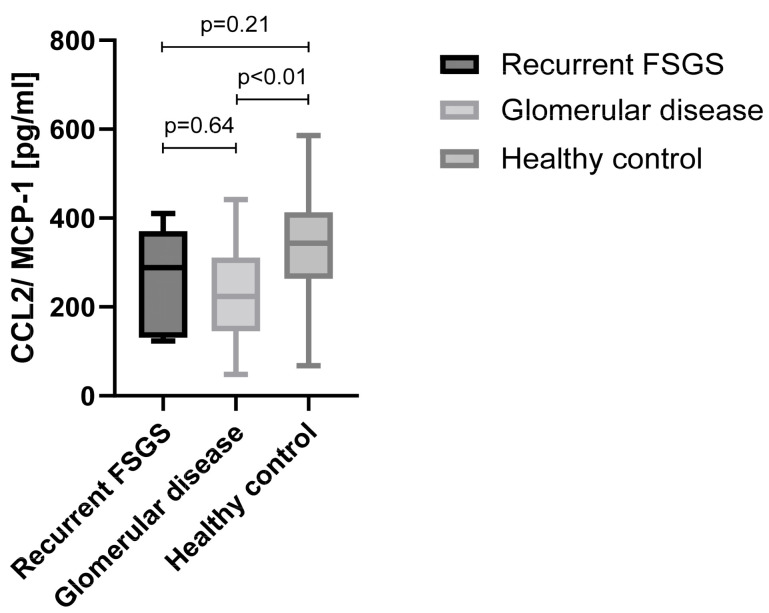
Plasma concentration of CCL2/MCP-1 in patients with FSGS recurrence after kidney transplantation compared to patients with glomerular disease and healthy individuals.

As an important representative of chemotaxis regulation, the CCL2/MCP-1 chemokine was measured by ELISA in our cohort. Although CCL2/MCP-1 exhibited significantly lower plasma levels in patients with glomerular diseases (237.6 ± 110.5 pg/mL; *n* = 36) compared to healthy subjects (341.5 ± 112.6 pg/mL; *p* < 0.01; *n* = 31), there was no difference between patients with FSGS recurrence after kidney transplantation (266.9 ± 118.0 pg/mL; *n* = 6) and those with glomerular diseases or healthy subjects (*p* = 0.64 and *p* = 0.21, respectively) (Figure 4).

## 4. Discussion

Recurrence of FSGS is a serious threat to the transplanted organ after kidney transplantation, affecting up to 60% of patients with primary FSGS as underlying disease [8]. Early treatment is key to preserve the transplant function, consisting of plasma exchange [38], immunoadsorption [39,40,41], rituximab [42], ofatumumab [43], abatacept [44], adrenocorticotropin hormone (ACTH) analogue gel [45], and high-dose steroids, among others. In general, patients with FSGS also have a significantly poorer graft survival than transplant recipients with other diseases [46]. It is therefore of particular importance to identify patients at risk of developing recurrence of disease in the allograft [47]. In this study, we therefore focused on investigating genetic predispositions and variations in immune response among patients experiencing recurrence of FSGS after kidney transplantation.

First, exome sequencing data of six patients with recurrence of FSGS after transplantation was analyzed. The aim was to identify clusters of potentially harmful gene variants playing a role in the pathogenesis of the disease. Our analyses led to the identification of 51 genes with SNPs causing missense mutations. Moreover, a significant finding was the high variation in the A2M protein, which subsequently became a focal point in our study. In particular, we detected variants of A2M at three patient-specific sites. The mutation Glu1165Gly, located near the TED domain, was classified as probably damaging with potential detrimental effect on protein function. This mutation was moreover associated with an altered protein aggregate formation, as was confirmed in our subsequent immunoblotting studies. A2M plays an important role in nephrotic syndrome [17] and FSGS [48], being upregulated in glomerular endothelial cells and showing elevated plasma levels in nephrotic patients to balance oncotic pressure when serum albumin is lost [15].

The presence of multiple gene variants, including the A2M mutation, underscores the complex genetic landscape of FSGS recurrence after transplantation. This suggests that while single gene mutations may confer increased risk, the phenotype of the disease may likely be multifactorial.

Building on the genetic findings, our subsequent studies utilized the Nephrocell server to investigate the cell-specific expression profiles of the identified 51 genes. This analysis was focused on the identification of possibly modified genes in FSGS. High expression of A2M, EXPH5, and PLEC could be verified in podocytes and endothelial cells of the glomerulus, indicating their potential role in the pathophysiology of FSGS.

This cellular focus opened the way for more comprehensive immunologic investigations, as abnormalities in certain cells can influence immune responses after transplantation. Our study was therefore extended to the area of immunological responses by performing a protein array screening. The aim of this screening was to explore the development of non-HLA-specific antibodies after kidney transplantation that could contribute to the recurrence of FSGS. A remarkable finding of this screening was the strong antibody binding to TRIM21—a gene involved in immune regulation and response to infections. TRIM21 functions as an intracellular Fc-gamma receptor and is a key regulator in the activation of cells, including podocytes [49]. It promotes the elimination of pathogens and the removal of autoantibodies and invading viruses [50]. Given the important immune function of podocytes within the nephron [51], changes in the protein activities could significantly affect the overall immune response, altering levels of circulating interleukins and chemokines. This could be particularly significant in the case of patient E with an IgG antibody against TRIM21, who experienced FSGS recurrence twice after two kidney transplantations and showed no response to any treatment intervention. It is noteworthy that this patient was found to have a heterozygous mutation in TRIM21 (Phe446Ile), which is classified as potentially dangerous, suggesting a link between genetic predisposition and immune response.

Moreover, a whole range of genes have already been associated with FSGS, several of which are expressed not only in podocytes or constituent structures of the glomerulus and therefore show different characteristics in manifestation from early onset to later manifestation [52,53,54,55,56]. In this context, the heterozygous missense mutation of EXPH5 was found in two of the patients of our study collective, of which the Gly1887Glu mutation was graded probably dangerous. Mutations of the EXPH5 gene, which is highly expressed in podocytes, have been reported to play an important role in epidermolysis bullosa [57], a disease that can be associated with congenital nephrotic syndrome and FSGS [58,59].

The above findings at the gene and protein level encouraged us to investigate the molecular mechanisms that could trigger recurrence of FSGS after transplantation. Hence, a detailed pathway and process enrichment analysis was performed [19], focusing on the downstream effects of the 51 identified gene variants. Thereby, at least 13 pathways were singled out in which mutated genes play an important role, in particular the IL-17 signaling and chemotaxis pathways.

IL-17 and CCL2/MCP-1, both considered factors for glomerular and podocyte pathologies [32], were measured by ELISA and correlated with clinical outcomes of the patients. Patients with recurrence of FSGS after kidney transplantation exhibited significantly elevated plasma levels of IL-17 compared to those with other glomerular diseases and healthy volunteers. In contrast, no differences in plasma levels of CCL2/MCP-1 were observed between patients with recurrence of FSGS, other glomerular diseases or to healthy controls.

A notable limitation is the small sample size of the study. Although our control groups included 45 patients with various glomerular diseases and 31 healthy control subjects, these numbers are still modest considering the variability and complexity of genetic and immunologic responses. Overall, this underscores the need for larger studies to validate and expand our understanding of recurrence of FSGS after transplantation.

## 5. Conclusions

In conclusion, patients with recurrent FSGS after kidney transplantation exhibited variants in 51 genes and showed modifications in gene expressions, particularly up-regulation of A2M, EXPH5, and PLEC in podocytes and glomerular endothelial cells. In addition, formation of non-HLA-specific antibodies, including TRIM21, could be delineated in patients with recurrence of FSGS. Finally, those with recurrence of FSGS in the transplanted organ exhibited significantly higher IL-17 serum levels compared to those with other glomerular diseases or healthy individuals.

This study expands the understanding of the genetic and immunologic mechanisms potentially driving the recurrence of FSGS after kidney transplantation. However, it does not determine in whom the disease recurs and why. Our investigation thereby underscores the multifactorial complexity of FSGS. The observation that single gene variants alone cannot entirely account for disease recurrence after transplantation highlights the need for a comprehensive, integrated approach. This study provides a framework for future research with larger cohorts and more comprehensive analyses of genetic and immunologic factors to deepen the understanding of the pathogenesis of FSGS and identify molecular targets. Continued scientific efforts are critical to prevent recurrence of disease, prolong graft survival, and improve the quality of life of transplant recipients.

## Figures and Tables

**Table 1:** Gene set with SNPs causing missense mutations.

A2M	CASP14	EXPH5	MUC5B	RGPD3
ABCA12	CATSPERB	FAM171B	NCOR2	RNF213
ABCC10	CD36	FER	OBSCN	SIL1
ABI3BP	DIDO1	GPR155	PKD1L1	SLIT2
AMOTL2	DNAH11	GPR179	PLEC	SORCS2
ANKRD11	DNAH3	KMT2C	PLXNA4	SSPO
ARHGEF4	DNAH8	MAPK15	PNKD	TAT
ARID1B	DPP4	MAPK6	PPP1R16A	TRIM21
ASPM	DPYS	MEGF6	PRRC2C	TRRAP
C1orf170	ERN1	MUC16	PTCH1	WHSC1L1
				ZIM3

Gene symbols for identified genes with missense mutations in six patients with recurrence of FSGS in the transplanted kidney; selected via the algorithm described above.

**Table 2:** Mutation pattern of the A2M gene in three of six patients with recurrence of FSGS after kidney transplantation.

ID	Transcript	Symbol	Function	pph2	Variant Alleles	Identifier	Omim
A	Lys691Asn	A2M	missense	benign	1	rs369389194	103,950
B	Glu1165Gly	A2M	missense	probably damaging	1		103,950
C	Leu18Arg	A2M	missense	benign	1		103,950

A2M = alpha-2 macroglobulin; pph2, PolyPhen-2 = prediction of functional effects of human non-synonymous single nucleotide polymorphisms (nsSNPs).

**Table 3:** Protein array screening using serum of patient E for IgG antibody detection. The strong serum IgG antibody binding to the TRIM21 protein (highlighted in bold) is particularly remarkable as patient E carries a non-synonymous Phe446Ile mutation classified as “potentially dangerous” in this gene.

ID	Intensity	MW Da
HIST1H3A	276	15,404
**TRIM21**	**205**	**54,170**
FAM84A	204	32,491
MAP9	196	74,234
DGKK	186	141,829
Nol3	121	22,629
CCDC70	118	28,767
HCLS1	105	54,014
ZYX	98	61,277
BSND	93	35,197
PPM1E	92	83,952
BAG3	87	61,595

Intensity refers to the fluorescence intensity. MW Da is the molecular weight in Dalton.

## Data Availability

The original contributions presented in the study are included in the article and Supplementary Material, further inquiries can be directed to the corresponding author.

## Supplementary Materials

The following supporting information can be downloaded at: https://www.mdpi.com/article/10.3390/diagnostics14151591/s1. File S1: Clinical course of patients with recurrence of FSGS after kidney transplantation; Figure S1: Structure of the native A2M monomer; Figure S2: A2M immunoblotting of serum without and with A2M variation; Figure S3: Gene expression analysis of A2M, EXPH5 and PLEC; Table S1: Pathway and process enrichment analysis; Table S2: Gene sets with mutations linked to the IL-17 pathway; Table S3: Gene sets with mutations associated with the chemotaxis signaling pathway.
