# Supplementary material for: Identification of Pathogenic Pathways for Recurrence of Focal Segmental Glomerulosclerosis after Kidney Transplantation
Source: Diagnostics (Basel). 2024 Jul 24;14(15):1591. doi: 10.3390/diagnostics14151591 (PMC11312181; doi:10.3390/diagnostics14151591)
Supplement: Supplementary file 1 [file diagnostics-14-01591-s001.zip › Supplement_Figure S1.pdf]

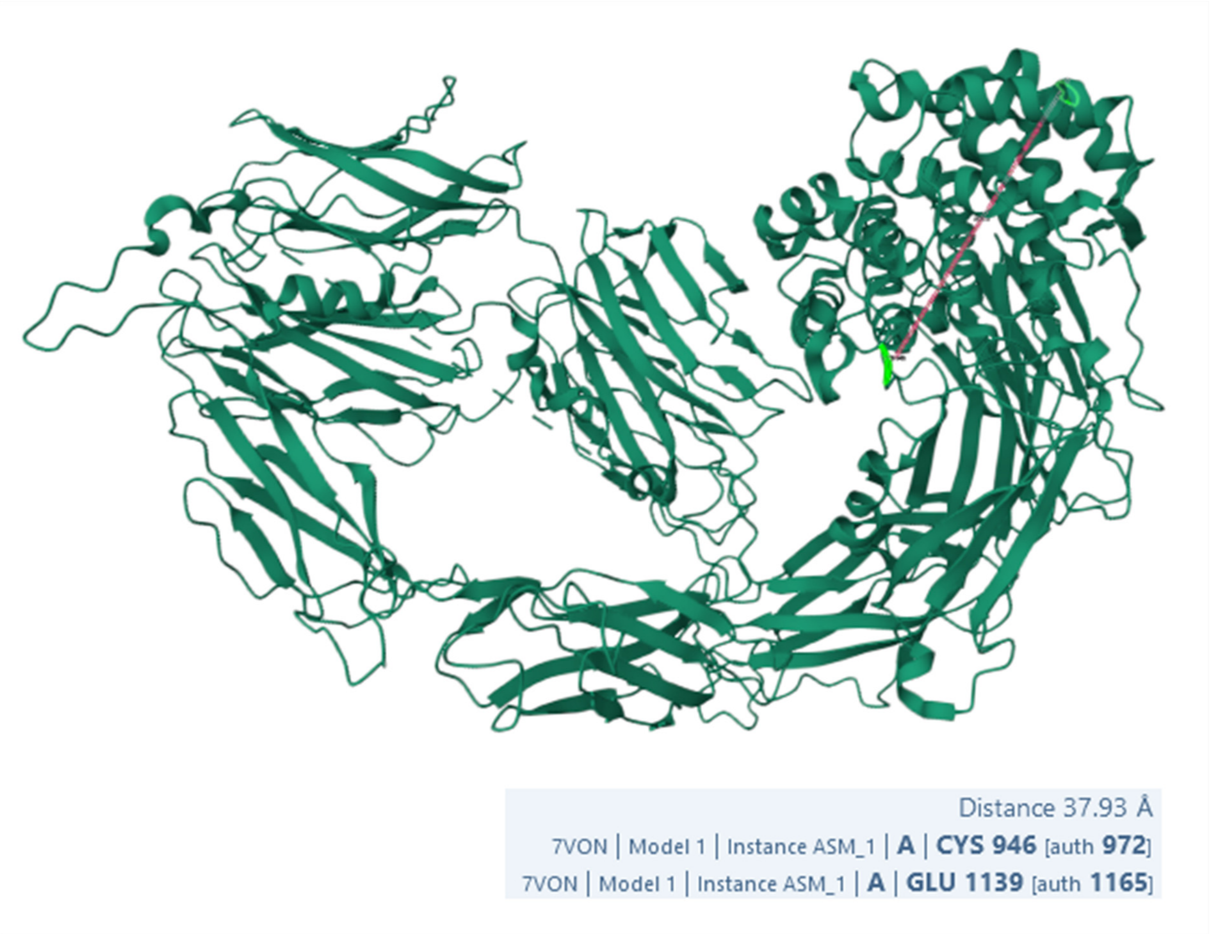

Supplementary Figure 1. According to the protein data bank (PDB) structure of the native alpha-2 macroglobulin monomer (7VON) the potential structure-altering property of the mutation results from its location about 38Å away from the cysteine of the thiol-ester domain 1 CGEQ 972-975 which is the functional unit of this protein.
