# Supplementary material for: Identification of Pathogenic Pathways for Recurrence of Focal Segmental Glomerulosclerosis after Kidney Transplantation
Source: Diagnostics (Basel). 2024 Jul 24;14(15):1591. doi: 10.3390/diagnostics14151591 (PMC11312181; doi:10.3390/diagnostics14151591)
Supplement: Supplementary file 1 [file diagnostics-14-01591-s001.zip › Supplement_Figure S2.pdf]

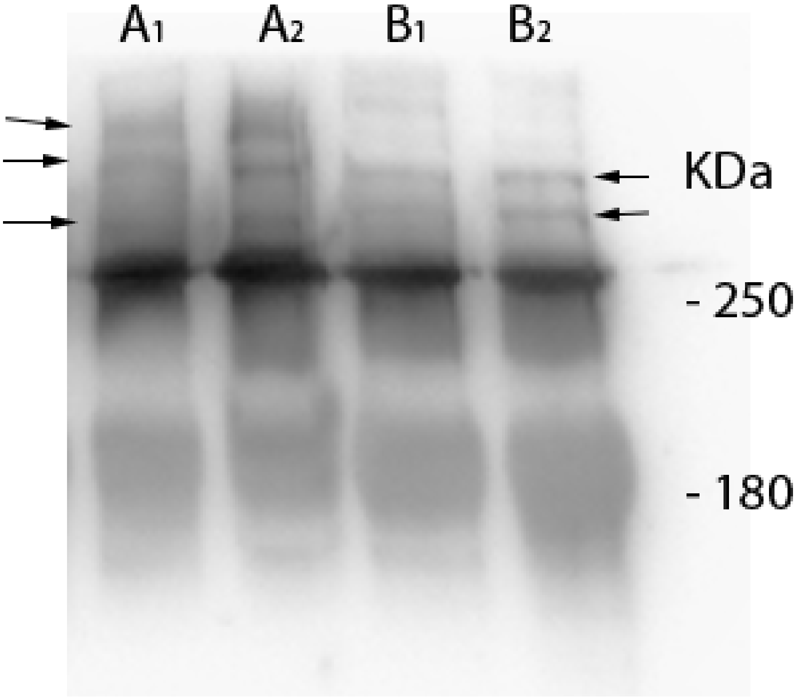

Supplementary Figure 2. A2M immunoblotting of serum without and with A2M variation. A_1 and A_2 depict a patient sample without A2M variation at two time points, while B_1 and B_2 show serum from patient B with the Glu1165Gly missense mutation at two time points. The arrows in columns A and B highlight a notable difference in the migratory pattern of the high molecular A2M aggregates.
