# Supplementary material for: Identification of Pathogenic Pathways for Recurrence of Focal Segmental Glomerulosclerosis after Kidney Transplantation
Source: Diagnostics (Basel). 2024 Jul 24;14(15):1591. doi: 10.3390/diagnostics14151591 (PMC11312181; doi:10.3390/diagnostics14151591)
Supplement: Supplementary file 1 [file diagnostics-14-01591-s001.zip › Supplement_Figure S3.pdf]

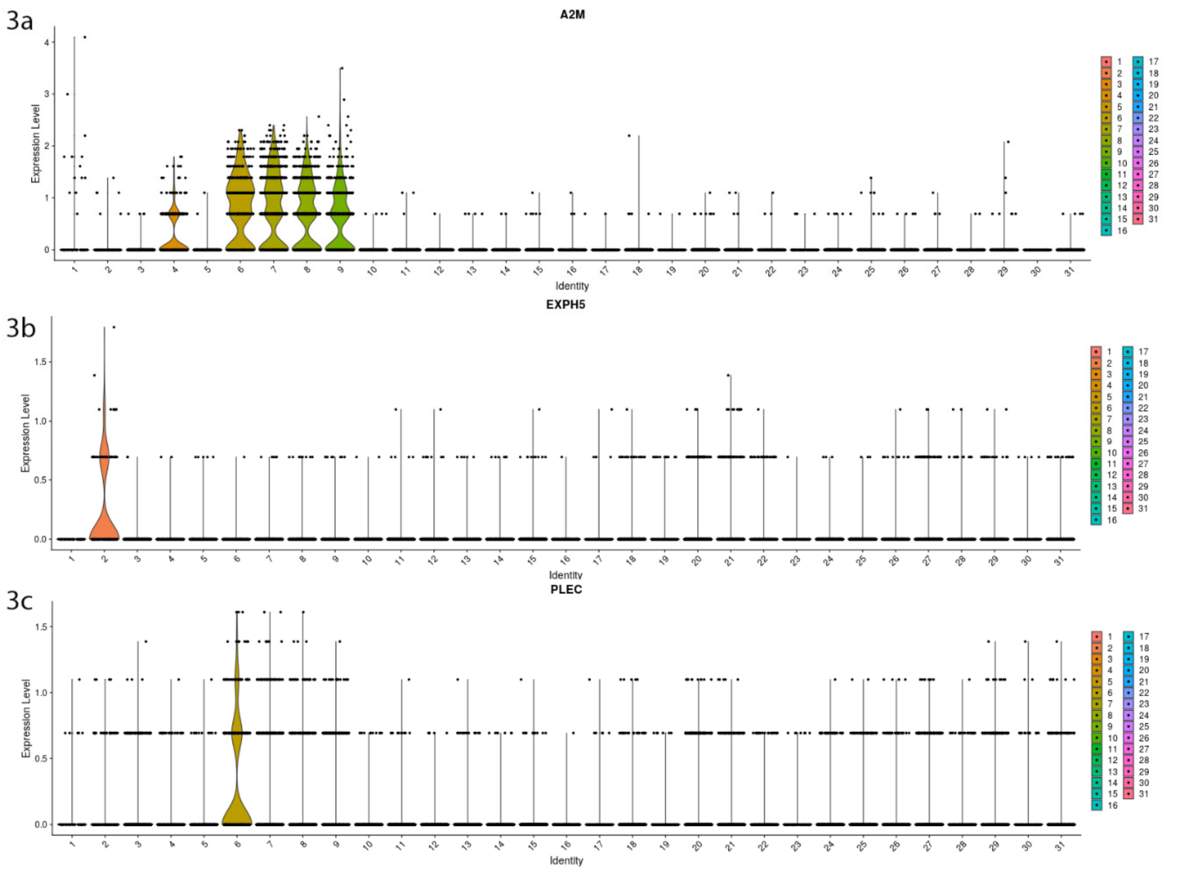

Supplementary Figure 3. The **A2M** gene transcripts taken from the Nephrocell Server is mainly expressed in the monocytes-cluster #4, the endothelial cell, arteriolar-cluster #6, the endothelial cell, peritubular-cluster #7, glomerular capillary endothelial cell-cluster #8 and in the vascular smooth muscle cell and mesangial-cluster #9 (**3a**). The **EXPH5** (exophilin 5) gene transcript is highly expressed in the podocyte-cluster #2 (**3b**) and the **PLEC** (plectin) gene transcript is highly expressed in the endothelial cell, arteriolar-cluster #6 (**3c**).
