# Supplementary material for: Identification of Pathogenic Pathways for Recurrence of Focal Segmental Glomerulosclerosis after Kidney Transplantation
Source: Diagnostics (Basel). 2024 Jul 24;14(15):1591. doi: 10.3390/diagnostics14151591 (PMC11312181; doi:10.3390/diagnostics14151591)

**<u>Supplementary File 1.</u>** <u>Clinical course of patients with recurrence of FSGS after kidney transplantation</u>

**Patient A:** The patient, diagnosed with FSGS at the age of 25, underwent unsuccessful initial treatment regimens involving steroid and cyclosporine A. Cyclophosphamide therapy and subsequent trial with immunoapheresis were discontinued due to lack of significant changes. The patient developed significant hyperlipidemia from extensive proteinuria and hypoalbuminemia, prompting the initiation of short-term lipidapheresis. Renal replacement therapy became necessary nine years after post diagnosis.

Years later, the patient received a renal allograft with a standard immunosuppressive regimen. Shortly after transplantation, substantial proteinuria recurred, and histologic findings were consistent with disease recurrence. Three months post-transplantat, a rise in serum creatinine prompted another biopsy, revealing transplant rejection according to BANFF criteria and changes indicative of recurrent FSGS. Pulse corticosteroid therapy was administered in response. Renal parameters remained stable thereafter, with serum creatinine between 2-3 mg/dl and proteinuria consistently ranging between 1.5-3g/d. Due to chronic deterioration, the patient lost the renal graft, resuming renal replacement therapy 16 years after transplantation.

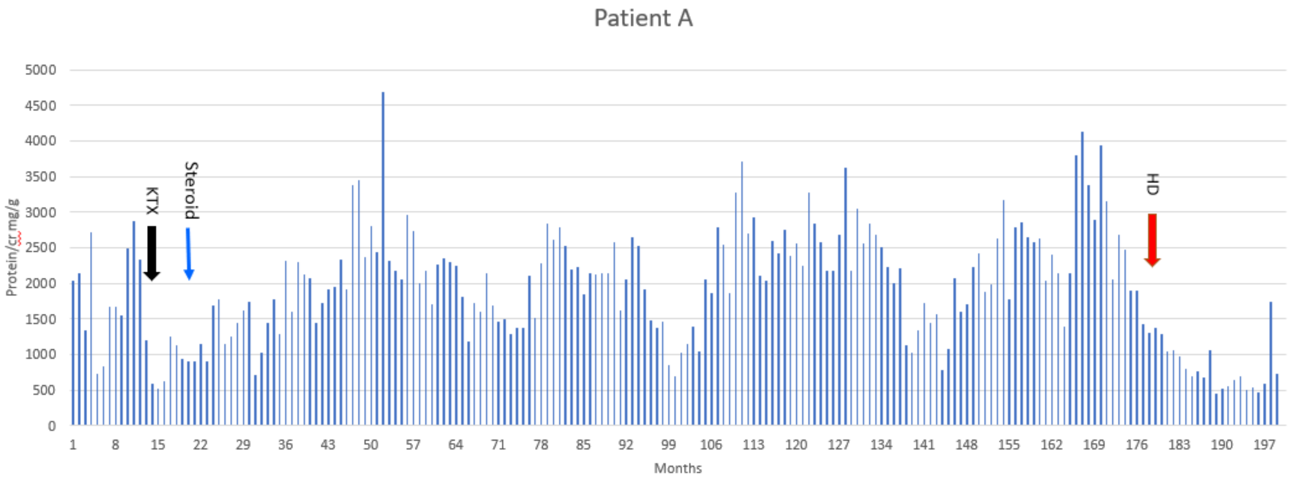

**Patient B:** The patient initiated hemodialysis due to chronic nephrotic syndrome in the course of FSGS. After kidney transplantation, the patient developed a BANFF IIa rejection, leading to treatment with antithymocyte globulin and steroids. Because of the additional diagnosis of impending FSGS recurrence, 9 plasma exchanges were performed. This resulted in partial remission, with protein excretion rates ranging between 1000 and 600 mg/24h. Subsequent plasma exchanges were followed by switch to rituximab. A graft biopsy one year after transplantation again revealed a BANFF Ia rejection episode, responsive to steroid treatment. Serum creatinine was stable at 1.3 mg/dl and the underlying disease was at remission.

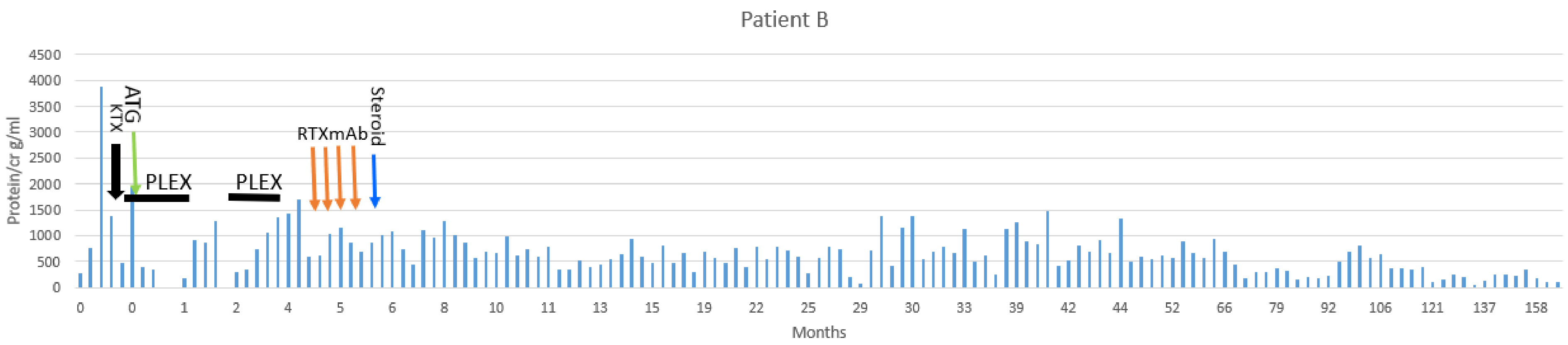

**Patient C:** The patient was diagnosed with FSGS at the age of nine presenting with nephrotic syndrome due to primary FSGS confirmed by renal biopsy. Subsequent to deteriorating renal function, the patient received a living donor transplant from his mother at the age of 21. At three months post-transplant, borderline rejection was diagnosed and successfully treated with steroids, resulting in a decrease of proteinuria and improved renal function parameters. Four years later, reoccurring nephrotic range proteinuria indicated disease recurrence. Rituximab therapy remained unsuccessful, but treatment with plasma exchange reduced proteinuria. Following close monitoring, subsequently immunoapheresis with a protein G column was initiated and continued for approximately two years, leading to disease remission.

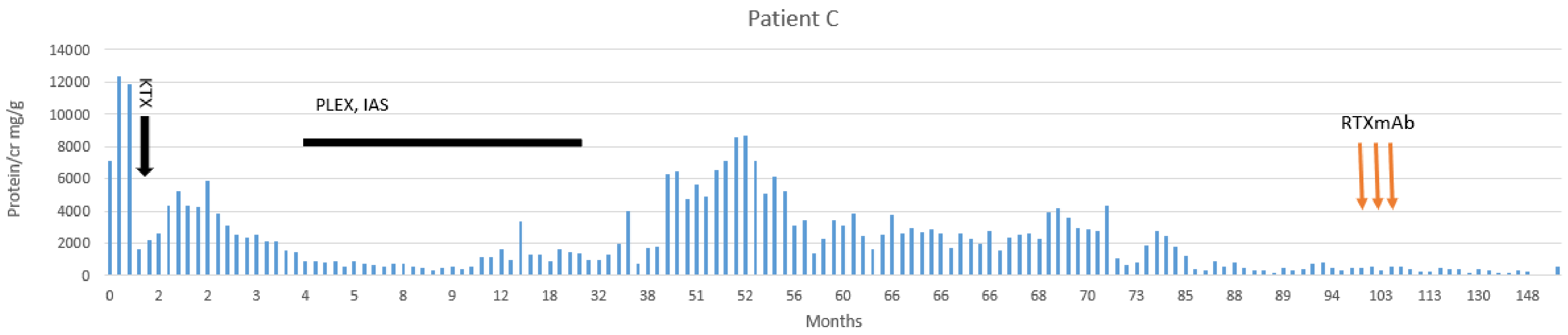

**Patient D:** The 57-years old patient was treated at the outpatient clinic for FSGS. Throughout the observation period, she received a series of immunosuppressants, including steroids, cyclophosphamide, cyclosporine A, tacrolimus, and mycofenolate mofetil. While this treatment initially reduced proteinuria, it did not lead to disease remission. Due to concomitant renal function decline, hemodialysis became necessary. Subsequently, kidney transplantation was performed, but weeks after transplantation, the patient was diagnosed with persistent proteinuria and recurrence of disease. Despite 2 cycles of plasma exchange and further treatment attempt with rituximab, remission was not achieved. While renal function parameters remained stable, patient continued to experience persistent proteinuria.

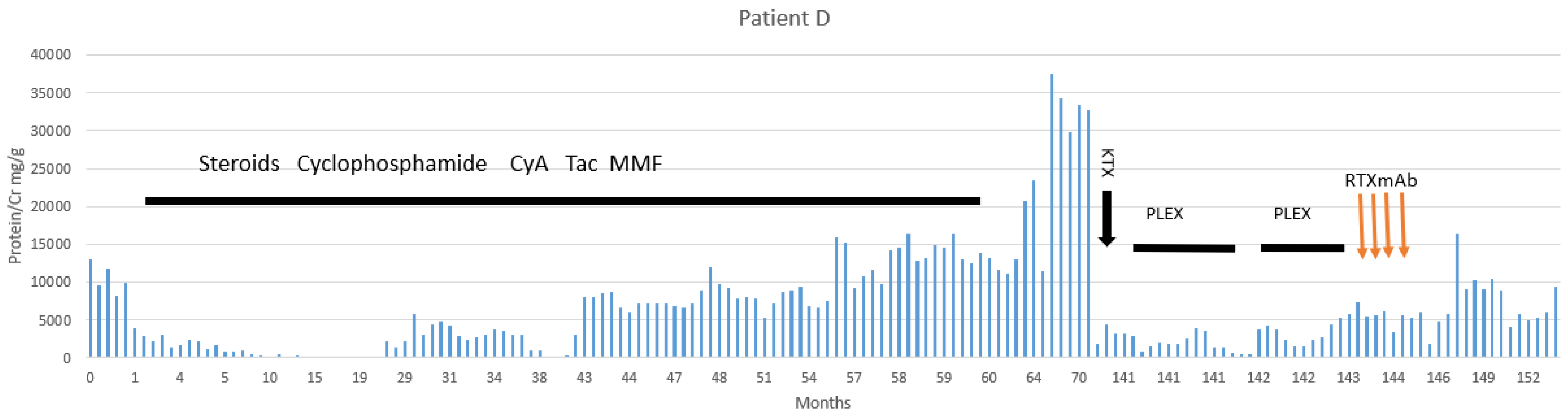

**Patient E:** The 23-years old patient underwent hemodialysis due to terminal chronic kidney disease resulting from focal segmental glomerulosclerosis (FSGS). After 5 years on dialysis, the patient received a renal allograft, but was immediately classified as primarily nonfunctioning for unknown reason. Retransplantation took place 8 months later, with a subsequent diagnosis of recurrence of FSGS in the renal allograft. After an unsuccessful treatment attempt with rituximab, immunoapheresis with protein G columns was initiated two months after kidney transplantation (KTX), followed by plasma exchange therapy. Repeated imunoadsorption and plasma exchange several months later proved ineffective against the resistant recurrence of FSGS. Ultimately, the patient required renal replacement therapy.

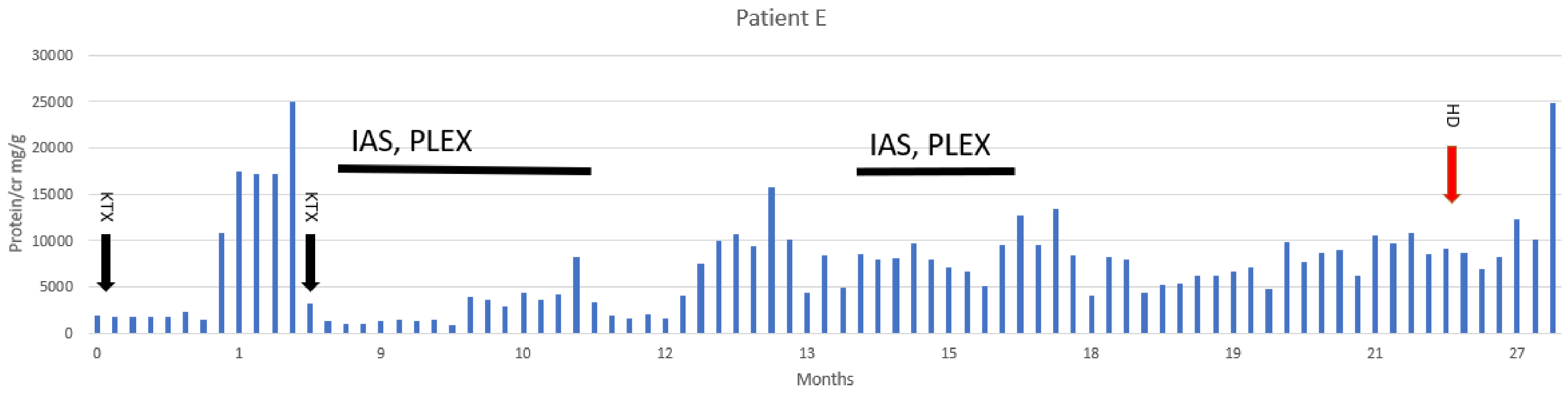

**Patient F:** The 34-years old patient, treated for FSGS as an outpatient at a referral hospital, underwent kidney allograft transplantation. Following the KTX, the patient required steroid therapy for borderline BANFF rejection and developed nephrotic range proteinuria with disease recurrence confirmed in a kidney biopsy. Despite an unsuccessful trial with abatacept, the underlying FSGS was managed with immunoapheresis using protein G column for 2 years. With stable kidney function and disease remission, immunoapheresis was discontinued and the patient received rituximab four times at a dose of 375 mg/m2. However, the patient suffered from recurrent urinary tract infections and worsening renal function, eventually, requiring hemodialysis due to end stage renal disease.

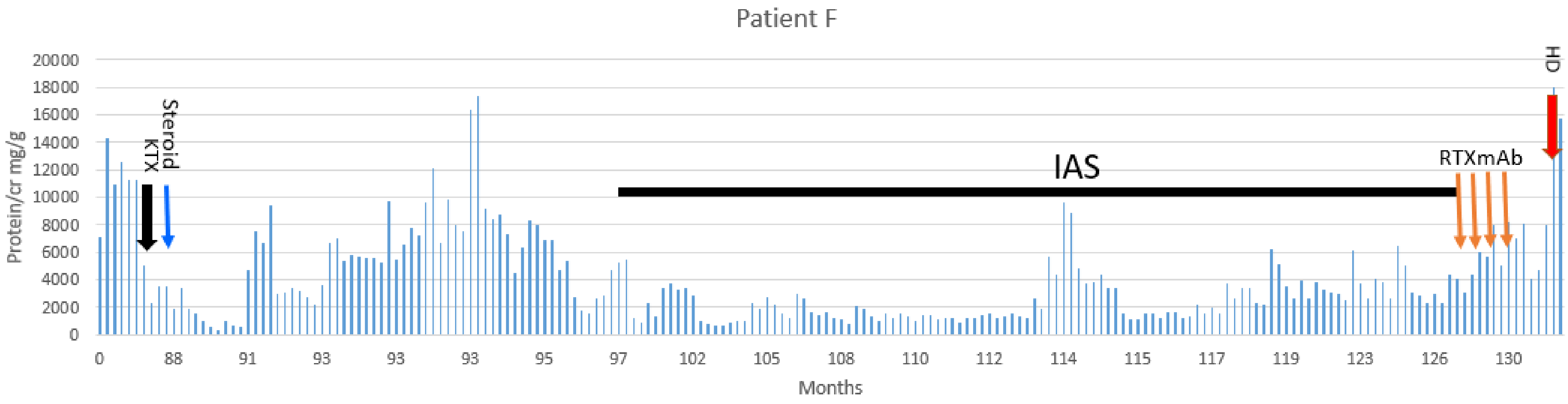

Supplement: Supplementary file 1 [file diagnostics-14-01591-s001.zip › Supplement_File S1.pdf]
