# Supplementary material for: Identification of Pathogenic Pathways for Recurrence of Focal Segmental Glomerulosclerosis after Kidney Transplantation
Source: Diagnostics (Basel). 2024 Jul 24;14(15):1591. doi: 10.3390/diagnostics14151591 (PMC11312181; doi:10.3390/diagnostics14151591)
Supplement: Supplementary file 1 [file diagnostics-14-01591-s001.zip › Supplement_Table S2.pdf]

Supplementary Table 2. Gene sets with mutations linked to the IL-17 pathway.

Patient ID	Protein	Description	Mutation
A	ERN1	benign	Asp558Glu
A	SLIT2	benign	Arg1113His
B	MAPK6	benign	Gln609Arg
B	MAPK15	probably dangerous	Arg260Trp
B	MAPK15	benign	Thr381Pro
B	ERN1	benign	His939Asn
C	OBSCN	possibly dangerous	Arg4558His
D	MUC5B	benign	Thr2980Met
D	CD36	probably dangerous	Thr136Ser
E	MUC5B	probably dangerous	Val1010Met
E	SLIT2	probably dangerous	Pro199Leu
F	OBSCN	benign	Val442Leu
