# Supplementary material for: Identification of Pathogenic Pathways for Recurrence of Focal Segmental Glomerulosclerosis after Kidney Transplantation
Source: Diagnostics (Basel). 2024 Jul 24;14(15):1591. doi: 10.3390/diagnostics14151591 (PMC11312181; doi:10.3390/diagnostics14151591)
Supplement: Supplementary file 1 [file diagnostics-14-01591-s001.zip › Supplement_Table S3.pdf]

Supplementary Table 3. Gene sets with mutations associated with the chemotaxis signaling pathway.

Patient ID	Protein	Description	Variant
A	AMOTL2	possibly damaging	Arg388Trp
A	SLIT2	benign	Arg1113His
A	PLXNA4	benign	Ser1891Asn
C	OBSCN	possibly damaging	Arg4558His
D	ASPM	benign	Pro1205Ala
D	AMOTL2	benign	Ala505Thr
D	ARID1B	probably damaging	Gln1385His
D	DPP4	possibly damaging	Thr312Ser
D	DNAH11	probably damaging	Ala87Gly
E	FER	probably damaging	Tyr430Cys
E	SLIT2	probably damaging	Pro199Leu
F	ARID1B	possibly damaging	Pro2225Leu
F	DPP4	probably damaging	Ala330Val
F	NCOR2	benign	Ser516Asn
F	OBSCN	benign	Val442Leu
F	PLXNA4	benign	Ser1891Asn
